# Screening prefrailty in Japanese community-dwelling older adults with daily gait speed and number of steps via tri-axial accelerometers

**DOI:** 10.1038/s41598-021-98286-0

**Published:** 2021-09-21

**Authors:** Naoto Takayanagi, Motoki Sudo, Yukari Yamashiro, Ippei Chiba, Sangyoon Lee, Yoshifumi Niki, Hiroyuki Shimada

**Affiliations:** 1 Tokyo Research Laboratories, Kao Corporation, 2-1-3 Bunka, Sumida-ku, Tokyo, 131-8501 Japan; 2 Department of Preventive Gerontology, Center for Gerontology and Social Science, National Center for Geriatrics and Gerontology, 7-430 Morioka, Obu, Aichi 474-8511 Japan

**Keywords:** Geriatrics, Quality of life

## Abstract

Prefrailty is an intermediate stage between non-frailty and frailty. It is associated with an increased risk of progression to frailty, which makes it important to screen older adults for prefrailty at an early stage. This study verified whether daily gait speed and number of steps measured using a tri-axial accelerometer could be used to identify prefrailty. In total, 1692 Japanese community-dwelling older adults were divided into robust (n = 1032) and prefrail (n = 660) groups based on the Kihon Checklist, which is a self-administered questionnaire. Both daily gait speed and number of steps were measured for two weeks using tri-axial accelerometers. We also calculated the area under the ROC curve and the cut-off values for these parameters. Our results showed that the cut-off value for daily gait speed was 106.3 cm/s, while that for number of steps was 6342.2. In addition, we found that the combined assessment of both cut-off values was a more effective way to screen older adults with prefrailty status compared to either parameter alone. This is also considered an effective way to reduce national expenditures for daily care assistance.

## Introduction

Frailty is a state of increased vulnerability due to the poor resolution of homeostasis following a stressor^1^ and the consequence of several chronic diseases, and promotes some of the changes that occur with age^2^. It is characterized by muscle weakness, cognitive decline, and loss of physical function, all of which are associated with increased disease risk^1^. Previous studies have reported that older adults with frailty are at an increased risk of falling, disability, hospitalization, and death^3,4^. This makes it essential to detect frailty in older adults to provide avenues to prevent continued deterioration.

To evaluate frailty status, Fried et al.^5^ proposed the Cardiovascular Health Study (CHS) frailty index. In this index, prefrailty is defined as an intermediate stage between non-frailty and frailty, which is also related to the start of functional disability^6^. Recent studies have reported that even prefrailty is associated with functional decline^6^, chronic comorbidity^7^, cognitive dysfunction^8^, and fall history^9^. Although prefrailty is associated with an increased risk of progression to frailty compared to non-frailty^10^, prefrailty is reportedly a reversible state^11,12^. As such, it seems to be an optimal target for clinical and/or behavioral interventions to slow or reverse worsening frailty. Older adults should therefore receive early screenings for prefrailty, which could support healthcare professionals and caregivers in preventing frailty.

Prefrailty can be assessed as a self-reported measure such as the Kihon Checklist (KCL), which consists of 25 questions designed to identify older adults at risk of requiring support^13^, or via clinical instruments such as a portable dynamometer, which measures strength^14^. Recently, Shimada et al. reported that older adults with prefrailty who showed slow gait speed in a laboratory setting were at an increased risk of future disability^15^. However, these measurements were taken in a limited environment under time constraints, both of which make it difficult to continuously measure relevant parameters.

Daily step measurements can be important indicators when screening older adults for prefrailty in a daily life context. When using an accelerometer, physical activity parameters can be measured continuously during gait in the free-living conditions of daily life, enabling the detection of prefrailty status in older adults outside the more limited constraints of laboratory environments. Recently, Razjouyan et al. reported that physical activity parameters, including the number of steps measured via accelerometers, were significant independent predictors of prefrailty status in older adults^16^. However, this study was conducted with a small number of participants (153 including 33 for frailty), and it is unclear whether this result can be applied to community-dwelling older adults on a large scale.

Previous studies have shown that gait speed can be assessed in free-living conditions (daily gait speed) via an accelerometer^17–19^. Although research has demonstrated an association between prefrailty and slow in-laboratory gait speed^15^, there is only a weak association between daily gait speed and in-laboratory gait speed^20^. Therefore, continued research is needed to clarify whether daily gait speed and/or in-laboratory gait speed are good parameters for screening prefrailty, with an additional focus on the connections between the two parameters.

This study verified whether daily gait speed and the number of steps measured via a tri-axial accelerometer could be used to identify prefrailty status as defined by the KCL. Bortone et al. conducted a systematic review of the relationship between gait parameters and frailty and stated that combining multiple gait parameters may enhance the prediction of frailty status and the classification of different frailty phenotypes^21^. In particular, no previous studies have examined the relationship between daily gait speed and prefrailty. In general, the number of steps represents gait quantity in the context of daily life, while daily gait speed represents the quality of gait in daily living. Therefore, we hypothesized that it would be better to screen older adults for this condition by combining the number of steps as a quantity of daily gait parameters with the daily gait speed as a quality of daily gait parameter.

## Results

Table 1 shows the demographic characteristics and gait parameters of the robust and prefrail groups. Significant differences were found in age (*p* < 0.001, d = 0.22), number of steps (*p* < 0.001, d = 0.28), and daily gait speed (*p* < 0.001, d = 0.25). Table 2 shows the diagnostic values of age and gait parameters, which significantly differed between the groups. The table also shows the area under the curve (AUC) and respective 95% confidence intervals (95% CI), sensitivity, and specificity for variables used as predictors of prefrailty. The AUC was observed for age (AUC, 0.558; 95% CI 0.529–0.586). In terms of the variables measured in free-living conditions, AUC was observed for the number of steps (AUC, 0.593; 95% CI 0.565–0.621) and daily gait speed (AUC: 0.567, 95% CI 0.539–0.595). The cut-off values for the variables were 68.5 years for age, 6342.2 steps/day for number of steps, and 106.3 cm/s for daily gait speed.

**Table 1:** Demographics and gait parameters of the robust and prefrail groups.

	All (n = 1692)	Robust (n = 1032)	Prefrail (n = 660)	Significant difference
Age (years)	70.0 ± 6.2	69.4 ± 5.9	70.8 ± 6.4	*p < 0.001 d = 0.22
Female sex, number (%)	1028 (60.8)	612 (59.3)	416 (63.0)	*Χ*^2^ = 2.346 *p* = 0.126 *V* = 0.048
Height (cm)	156.5 ± 8.5	156.7 ± 8.3	156.2 ± 8.7	*p* = 0.332 d = 0.05
Weight (cm)	57.5 ± 10.0	57.7 ± 9.8	57.3 ± 10.4	*p* = 0.444 d = 0.04
BMI (kg/m^2^)	23.4 ± 3.1	23.4 ± 2.9	23.4 ± 3.4	*p* = 0.904 d = 0.01
Number of steps (steps/day)	6591.7 ± 2982.6	6914.5 ± 2883.1	6087.0 ± 3066.6	*p < 0.001 d = 0.28
Daily gait speed (cm/s)	110.6 ± 22.6	112.8 ± 23.2	107.2 ± 21.3	*p < 0.001 d = 0.25

Data are shown as means ± SDs. Unpaired-*t* tests or chi-square tests were conducted between groups.

BMI = body mass index.

*p < 0.05, d > 0.20.

**Table 2:** Diagnostic values of age and gait parameters for screening prefrailty.

	AUC (95% CI)	Sensitivity	Specificity	Cut-off values
Age (years)	0.558 (0.529–0.586)	56.8%	47.1%	68.5
Number of steps (steps/day)	0.593 (0.565–0.621)	62.9%	53.4%	6342.2
Daily gait speed (cm/s)	0.567 (0.539–0.595)	55.8%	54.5%	106.3

AUC = Area under the curve, CI = confidence interval.

Based on the cut-off value of the number of steps, all participants were classified into low steps (with number of steps < 6342.2 steps/day; n = 796) and high steps (with number of steps ≥ 6342.2 steps/day; n = 896) groups. Based on the cut-off value of daily gait speed, all participants were classified into low speed (with daily gait speed < 106.3 cm/s; n = 854) and high speed (with daily gait speed ≥ 106.3 cm/s; n = 838) groups. Table 3 shows the association of the number of steps or daily gait speed with prefrailty based on binomial logistic regression models. After adjusting for covariates by age, sex, and body mass index (BMI), for the number of steps model, the high-steps group had significantly lower odds ratios for prefrailty than the low-steps group (odds ratio: 0.556, 95% CI 0.452–0.684, *p* < 0.001). For the daily gait speed model, the high-speed group had significantly lower odds ratios for prefrailty than the low-speed group (odds ratio, 0.738; 95% CI 0.599–0.909, *p* = 0.004).

**Table 3:** Association of number of steps or daily gait speed with prefrailty in the binomial logistic regression models.

Model	Group	Crude		Adjusted^+^	
		Odds ratio (95% CI)	Significant difference	Odds ratio (95% CI)	Significant difference
Number of steps	Low Steps (n = 796)	Reference	*p < 0.001	Reference	*p < 0.001
	High Steps (n = 896)	0.515 (0.422–0.629)		0.556 (0.452–0.684)	
Daily gait speed	Low Speed (n = 854)	Reference	*p < 0.001	Reference	*p = 0.004
	High Speed (n = 838)	0.664 (0.545–0.808)		0.738 (0.599–0.909)	

^+^Adjusting variables were age, sex, and body mass index (BMI). CI, confidence interval; *p < 0.05.

Low Steps: Participants with a number of steps of < 6342.2 steps/day.

High Steps: Participants with a number of steps of ≥ 6342.2 steps/day.

Low Speed: Participants with a daily gait speed of < 106.3 cm/s.

High Speed: Participants with a daily gait speed of ≥ 106.3 cm/s.

Furthermore, based on both cut-off values (number of steps and daily gait speed), all participants were classified into the following four groups:

- Low Steps & Low Speed: Participants with their number of steps < 6342.2 steps/day and daily gait speed < 106.3 cm/s (n = 546).
- Low Steps & High Speed: Participants with their number of steps < 6342.2 steps/day and daily gait speed ≥ 106.3 cm/s (n = 350).
- High Steps & Low Speed: Participants with their number of steps ≥ 6342.2 steps/day and daily gait speed < 106.3 cm/s (n = 292).
- High Steps & High Speed: Participants with their number of steps ≥ 6342.2 steps/day and daily gait speed ≥ 106.3 cm/s (n = 504).

Table 4 shows the association between the combined number of steps and daily gait speed with prefrailty based on binomial logistic regression models. Compared with the low-steps & low-speed group, significantly lower odds ratios for prefrailty were observed in all other groups, even after adjusting for covariates by age, sex, and BMI (odds ratio: 0.468–0.751). In particular, the high-steps & high-speed group had the lowest odds ratio compared to the low-steps & low-speed group (odds ratio: 0.468, 95% CI 0.356–0.616, *p* < 0.001).

**Table 4:** Association of combined number of steps and daily gait speed with prefrailty in the binomial logistic regression models.

Model	Group	Crude		Adjusted^+^	
		Odds ratio (95% CI)	Significant difference	Odds ratio (95% CI)	Significant difference
Number of steps and Daily gait speed	Low Steps & Low Speed (n = 546)	Reference		Reference	
	Low Steps & High Speed (n = 350)	0.696 (0.531–0.913)	*p = 0.009	0.751 (0.567–0.994)	*p = 0.045
	High Steps & Low Speed (n = 292)	0.493 (0.367–0.664)	*p < 0.001	0.527 (0.390–0.712)	*p < 0.001
	High Steps & High Speed (n = 504)	0.423 (0.328–0.545)	*p < 0.001	0.468 (0.356–0.616)	*p < 0.001

^+^Adjusted variables were age, sex, and body mass index (BMI). CI = confidence interval; *p < 0.05.

Low Steps & Low Speed: Participants with a number of steps of < 6,342.2 steps/day and daily gait speed < 106.3 cm/s.

Low Steps & High Speed: Participants with a number of steps of < 6,342.2 steps/day and daily gait speed ≥ 106.3 cm/s.

High Steps & Low Speed: Participants with a number of steps of ≥ 6,342.2 steps/day and daily gait speed < 106.3 cm/s.

High Steps & High Speed: Participants with a number of steps ≥ 6342.2 steps/day and daily gait speed ≥ 106.3 cm/s.

**Figure 1:**
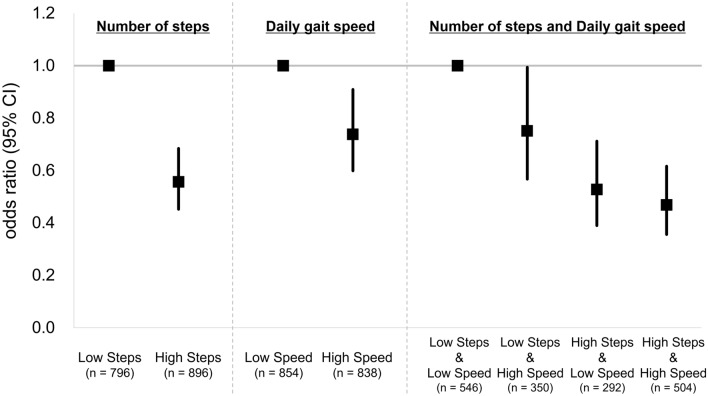
Odds ratios (95% CI) of the three models (number of steps, daily gait speed, and combined two parameters) for prefrailty based on the cut-off values for number of steps and daily gait speed. ^+^Adjusted variables were age, sex, and body mass index (BMI). CI = confidence interval.

Figure 1 shows the odds ratios and 95% CIs of the three models (number of steps, daily gait speed, and combined two parameters) for prefrailty based on the cut-off values for the number of steps and daily gait speed.

## Discussion

This study verified whether daily gait speed and the number of steps measured using a tri-axial accelerometer could be used to identify prefrailty status in Japanese community-dwelling older adults. We hypothesized that it would be better to screen older adults for prefrailty status by combining two daily gait parameters rather than using a single parameter alone. Our results showed clear cut-off values for both daily gait speed and number of steps, with both parameters also showing a protective association with prefrailty, even when values were lower than the cut-off values.

The total KCL score is widely used to classify frailty status in Japan. It was developed to identify older individuals at risk of requiring care or support within the Japanese long-term care insurance system, independent of the concept of frailty^13^. A systematic review by Sampaio et al.^22^ confirmed KCL as a reliable tool for predicting general frailty in older adults. In this study, 39.0% of participants had a prefrailty status according to the KCL criteria (1,032 as robust and 660 as prefrail). In a previous study among Japanese older adults, 35.4% of participants had prefrailty status (2,962 as robust and 1,625 as prefrail)^23^, similar to the percentages reported in related studies across Japan. Furthermore, this study found significant differences in age between the robust and prefrail groups. In this regard, multiple studies have reported a relationship between frailty status and age^5,24^. Based on these factors, we considered that the distribution of participants in this study met an equivalent standard.

In this study, we found a significant difference between the robust and prefrail groups in the number of steps. Notably, Theou et al. reported that the number of steps measured using an accelerometer is strongly correlated with frailty^25^. Furthermore, few cases have demonstrated an association between the number of steps and prefrailty. Park et al. found that it was necessary to walk 7,000–8,000 steps/day to prevent transition to the state of sarcopenia^26^, which is recognized as a major health problem among older adults. In fact, it is associated with serious health consequences in terms of frailty^27^. In this study, the average number of steps in the robust group was 6,914.5 ± 2,883.1 steps/day, but that for the prefrail group was 6,087.0 ± 3,066.6 steps/day. Compared with individuals in the robust group, those in the prefrail group may have therefore lacked the physical activity needed to prevent deterioration of sarcopenia.

We also found a significant difference in daily gait speed between the two groups. Although a previous study used a GPS to report on outdoor activity speed, only 28 participants were involved (low frailty index, 13; intermediate frailty index, 9; high frailty index, 6), with no clear relationships between groups^25^. To our knowledge, the present study is the first to report on the relationship between daily gait speed as measured using a tri-axial accelerometer and prefrailty status among a large number of older adults. Measured via an accelerometer, earlier results obtained from 1,965 older adults showed that daily gait speed declined with age^20^. This suggests that decreased physical functioning, which affects muscle strength in the lower limbs, may be an important associated factor. Further, participants with prefrailty status showed a noticeable decrease in physical functioning as a result of aging.

In this study, the AUC was calculated for variables that significantly differed between the robust and prefrail groups. The lowest AUC was observed for age (0.558), while the AUCs observed for the number of steps and daily gait speed were 0.593 and 0.568, respectively. Razjouyan et al. reported that physical activity parameters, including number of steps, were significant independent predictors of prefrailty status in older adults, with a high AUC > 0.7 (particularly 0.89 ± 0.02 for number of steps)^16^. This discrepancy may be due to several factors. First, the previous study included 153 participants (including 33 with frailty), while this study included 1692 participants. Second, the number of steps for the robust group in the previous study was approximately 12,200 steps/day, much higher than the results of the present study for the robust group (6914.5 ± 2883.1 steps/day). These results imply that participants in the previous study may have been a population with limited activity. In addition, many older adults with non-frailty status who were also not active may have been included in this study. This can be a valuable insight that measures the relationship between daily gait parameters (number of steps and daily gait speed) and prefrailty in community-dwelling older adults on a large scale.

Based on the cut-off values for the number of steps and daily gait speed, three binomial logistic regression models (number of steps, daily gait speed, and combined two parameters) for prefrailty were constructed. For the two single parameter models, the odds ratios and 95% CIs of the high-steps group (odds ratio: 0.556, 95% CI 0.452–0.684) and high-speed group (odds ratio: 0.738, 95% CI 0.599–0.909) were obtained compared with each reference group. These results suggest that the number of steps model can better predict prefrailty status in older adults compared with the daily gait speed model. The 25-item KCL questionnaire^13^ used in this study includes questions about the frequency and intention of going out, such as “Do you go out at least once a week?”, “Do you go out less frequently compared to last year?”, “Do you go out by bus or train by yourself?”, “Do you sometimes visit your friends?”, and “Do you go shopping to buy daily necessities by yourself?”. Harada et al. reported that outdoor time was significantly associated with the number of steps and was indirectly associated with physical function^28^. Therefore, it is likely that the model using the number of steps was more strongly associated with prefrailty, as defined by the KCL in this study.

Furthermore, for the model combining the two cut-off values for the number of steps and daily gait speed, the odds ratios and 95% CIs of the low-steps & high-speed group (odds ratio: 0.751, 95% CI 0.567–0.994) and high-steps & low-speed group (odds ratio: 0.527, 95% CI 0.390–0.712) were obtained compared with the reference group (i.e., low-steps & low-speed group). In particular, the lowest odds ratios and 95% CIs of the high-steps & high-speed group were obtained (odds ratio: 0.468, 95% CI 0.356–0.616) compared with the reference group. Interestingly, this was also the lowest odds of prefrailty compared with those of the two single parameter models (i.e., only daily gait speed or the number of steps). This result suggests that the combined use of both daily gait parameters can facilitate the screening process of prefrailty status in older adults. Bortone et al. performed a systematic review of the relationship between gait parameters and frailty and stated that the combination of various gait parameters may enhance the prediction of frailty status and the classification of different frailty phenotypes^21^. The results of this study, which used two daily gait parameters, support the importance of combining multiple parameters in classifying prefrail status in older adults.

Beginning with the first formal studies on frailty, the associated physical aspects have attracted much attention. In recent years, however, studies have also begun to focus on evaluation methods, including psychological and social aspects. Rockwood et al. proposed an evaluation method (frailty index) based on 70 items, such as activities in daily life and psychosocial risk factors^29^. The KCL used in this study consists of 25 questions^13^, with an evaluation principle similar to that proposed by Rockwood et al.^29^. Previous studies also found that the number of steps is related to mental states such as depressive mood^30^ and outdoor time^28^. Decreases in the number of steps are thus likely to affect psychosocial aspects, because such reductions are largely influenced by individual willingness. Decreased daily walking speed is also likely to affect physical aspects, including decreased muscle strength and muscle mass in the lower limbs due to aging^20^. From this perspective, prefrailty can be predicted from multiple viewpoints involving a variety of physical and psychosocial aspects by combining the cut-off values of two parameters (number of steps and daily gait speed).

This study had some limitations. An important limitation is that the study focused on older adults living in Takahama City, Aichi, Japan. The cut-off values for the number of steps and daily gait speed are likely to change depending on the country or region. Therefore, to increase generalizability, future studies should compare national, regional, and/or cultural differences based on similar measurements. Second, we did not consider the types of shoes worn during the gait measurements. Third, the accelerometer contained an LCD screen so that participants could see their step counts. Therefore, the amount of activity implemented during the assessment period may have been higher than that implemented on ordinary free-living days^31^.

This study verified whether daily gait speed and steps measured using a tri-axial accelerometer could be used to identify prefrailty status. Our results showed a cut-off value for daily gait speed of 106.3 cm/s, while that for number of steps was 6342.2. We also found that the combined use of both cut-off values constituted a more effective way to screen older adults for prefrailty status compared to the use of either parameter alone. These screenings are also considered effective ways to reduce national expenditures on daily care assistance.

## Methods

### Participants

This study used data obtained from the Takahama Study of Health Promotion for Older Adults, which was conducted from September 2015 to June 2016. It is part of the National Center for Geriatrics and Gerontology Study of Geriatric Syndromes (NCGG-SGS), which is a cohort study aimed at establishing a screening system for geriatric syndromes^32^. The inclusion criteria for this study were as follows: age ≥ 60 years and residence in Takahama City, Aichi, Japan. In total, 4,072 community-dwelling older adults were included, all of whom agreed to wear accelerometers and provided written informed consent by reading and signing a consent form approved by the institutional review board. This study was conducted in accordance with the guidelines of the Declaration of Helsinki. The study protocol was approved by the Research Ethics Committee of the National Center for Geriatrics and Gerontology (Approval Number 1440–2).

### Daily data collection

Participants were instructed to wear tri-axial accelerometers (HW-100, Kao Corporation, Tokyo, Japan) on their waists at all times while awake, except during swimming or bathing, and to maintain their usual activities. They were also instructed to visit 1 of 75 designated places in Takahama City once over a period of 30–40 days so that their accelerometer data could be downloaded onto a tablet computer through a near field communication (NFC) system (RC-380, Sony Corporation, Tokyo, Japan). Designated places included public facilities, gyms, drug stores, cafeterias, and beauty salons, thus making it easy for participants to visit a range of preferred locations. The physical activity data collected through this system were managed on a server.

### Daily gait speed and number of steps measurement

Daily gait speed and number of steps were measured using an accelerometer (HW-100) to ensure continuous monitoring during daily living^20^. Daily gait speed was specifically calculated by composite acceleration during one gait cycle, as obtained by the tri-axial acceleration measurements, following an accuracy evaluation outlined in previous research^20^. The accelerometer also measured wearing time. For inclusion in this study, a valid day was defined as any on which the accelerometer was worn for ≥ 10 hours^33,34^.

### Determination of prefrailty status

A self-administered questionnaire survey was conducted using the Kihon Checklist (KCL), which consists of 25 questions designed to identify older adults at risk of requiring support. Satake et al.^13^ reported that total KCL scores were correlated with the number of frailty phenotypes according to the Cardiovascular Health Study (CHS) criteria, which is the most widely accepted screening tool for frailty. Following procedures implemented in a previous study, we defined scores of ≥ 8 as frailty, 4–7 as prefrailty, and 0–3 as robust. In this study, frail participants were excluded from the analysis.

### Data analysis

Individuals were excluded from participation if they (1) did not visit the designated places within 60 days of the date of instruction to begin wearing the accelerometer (n = 1148), (2) were unable to meet the criteria for obtaining accelerometer data (n = 958) (i.e., wearing the accelerometer on their waist for a total duration of ≥ 7 days, for ≥ 10 h/day, during the first 14 days after the day they began wearing the accelerometer); (3) had missing values on variables from the total KCL score (n = 37); (4) were defined as frail based on the KCL (n = 237). After vetting for these conditions, 1692 participants (41.6%) were included in the final analysis. Their average wearing day was 11.89 ± 2.21 days, and average wearing time was 14.21 ± 1.89 h/day.

### Statistics

Unpaired *t-tests* and chi-squared tests were conducted to analyze the demographics (age, sex, height, weight, and BMI) and gait parameters (number of steps, daily gait speed) of the robust and prefrail groups. Sensitivity, specificity, and cut-off values for variables that significantly differed between these groups were identified using receiver operating characteristic (ROC) curves to predict prefrailty status. The area under the ROC curve (AUC) was also calculated. The cut-off values were obtained from the maximal Youden’s index (calculated as sensitivity + specificity − 1) and the best combination of sensitivity and specificity. The associations between prefrailty and each daily gait parameter (number of steps or daily gait speed) were examined using binomial logistic regression models. Odds ratios and 95% confidence intervals (95% CI) of prefrailty compared with the low-steps or low-speed group, classified based on the cut-off values, were calculated in the crude and adjusted models for each daily gait parameter (adjusting variables were age, sex, and BMI). Furthermore, the associations between prefrailty and the four groups classified based on both cut-off values (number of steps and daily gait speed) were examined using binomial logistic regression models. The odds ratios and 95% CIs of prefrailty compared with the low number of steps and low daily gait speed group (low-steps & low-speed group) were calculated in the crude and adjusted models for each group (adjusting variables were age, sex, and BMI). Differences in means were considered statistically significant when *p* values were less than 0.05, *d* values were greater than 0.20, and *V* values were greater than 0.10^35^. All statistical analyses were conducted using the IBM SPSS statistical software package (IBM SPSS Statistics Version 23, SPSS Inc., Chicago, IL, USA).
