# Author Correction: Screening prefrailty in Japanese community‑dwelling older adults with daily gait speed and number of steps via tri‑axial accelerometers

**DOI:** 10.1038/s41598-021-99782-z

**Published:** 2021-10-05

**Authors:** Naoto Takayanagi, Motoki Sudo, Yukari Yamashiro, Ippei Chiba, Sangyoon Lee, Yoshifumi Niki, Hiroyuki Shimada

**Affiliations:** 1 Tokyo Research Laboratories, Kao Corporation, 2‑1‑3 Bunka, Sumida‑ku, Tokyo, 131‑8501 Japan; 2 Department of Preventive Gerontology, Center for Gerontology and Social Science, National Center for Geriatrics and Gerontology, 7‑430 Morioka, Obu, Aichi 474‑8511 Japan

Correction to: *Scientific Reports* https://doi.org/10.1038/s41598-021-98286-0, published online 21 September 2021

The original version of this Article contained errors in data analysis and number of participants. As the result, in the Methods section, under the subheading ‘Data analysis’,

“Individuals were excluded from participation if they (1) did not visit the designated places within 60 days of the date of instruction to begin wearing the accelerometer (n = 1148), (2) were unable to meet the criteria for obtaining accelerometer data (n = 958) (i.e., wearing the accelerometer on their waist for a total duration of ≥ 7 days, for ≥ 10 h/day, during the first 14 days after the day they began wearing the accelerometer); (3) had missing values on variables from the total KCL score (n = 37); (4) were defined as frail based on the KCL (n = 237), and/or (5) were certified as needing care within the Japanese long-term care insurance system (n = 4). After vetting for these conditions, 1929 participants (41.6%) were included in the final analysis. Their average wearing day was 11.89 ± 2.21 days, and average wearing time was 14.21 ± 1.89 h/day.”

now reads:

“Individuals were excluded from participation if they (1) did not visit the designated places within 60 days of the date of instruction to begin wearing the accelerometer (n = 1148), (2) were unable to meet the criteria for obtaining accelerometer data (n = 958) (i.e., wearing the accelerometer on their waist for a total duration of ≥ 7 days, for ≥ 10 h/day, during the first 14 days after the day they began wearing the accelerometer); (3) had missing values on variables from the total KCL score (n = 37); (4) were defined as frail based on the KCL (n = 237). After vetting for these conditions, 1692 participants (41.6%) were included in the final analysis. Their average wearing day was 11.89 ± 2.21 days, and average wearing time was 14.21 ± 1.89 h/day.”

The original Article has been corrected.

